# Loss of heterozygosity at the *ATBF1-A *locus located in the 16q22 minimal region in breast cancer

**DOI:** 10.1186/1471-2407-8-262

**Published:** 2008-09-16

**Authors:** Kazuharu Kai, Zhenhuan Zhang, Hiroko Yamashita, Yutaka Yamamoto, Yutaka Miura, Hirotaka Iwase

**Affiliations:** 1 Department of Breast and Endocrine Surgery, Faculty of Medical and Pharmaceutical Science, Kumamoto University, Kumamoto, Japan; 2 Department of Breast and Endocrine Surgery, Nagoya City University, Medical School, Nagoya, Japan; 3 Department of Bioregulation Research, Nagoya City University Medical School, Nagoya, Japan

## Abstract

**Background:**

Loss of heterozygosity (LOH) on the long arm of chromosome 16 is one of the most frequent genetic events in solid tumors. Recently, the *AT-motif binding factor 1 *(*ATBF1*)-*A *gene, which has been assigned to chromosome 16q22.3-23.1, was identified as a plausible candidate for tumor suppression in solid tumors due to its functional inhibition of cell proliferation and high mutation rate in prostate cancer. We previously reported that a reduction in *ATBF1-A *mRNA levels correlated with a worse prognosis in breast cancer. However, the mechanisms regulating the reduction of *ATBF1-A *mRNA levels (such as mutation, methylation in the promoter region, or deletion spanning the coding region) have not been fully examined. In addition, few studies have analyzed LOH status at the *ATBF1-A *locus, located in the 16q22 minimal region.

**Methods:**

Profiles of *ATBF1-A *mRNA levels that we previously reported for 127 cases were used. In this study, breast cancer specimens as well as autologous blood samples were screened for LOH using 6 polymorphic microsatellite markers spanning chromosome band 16q22. For mutational analysis, we selected 12 cases and analyzed selected spots in the *ATBF1-A *coding region at which mutations have been frequently reported in prostate cancer.

**Results:**

Forty-three cases that yielded clear profiles of LOH status at both D16S3106 and D16S3018 microsatellites, nearest to the location of the *ATBF1-A *gene, were regarded as informative and were classified into two groups: LOH (22 cases) and retention of heterozygosity (21 cases). Comparative assessment of the *ATBF1-A *mRNA levels according to LOH status at the *ATBF1-A *locus demonstrated no relationship between them. In the 12 cases screened for mutational analysis, there were no somatic mutations with amino acid substitution or frameshift; however, two germ line alterations with possible polymorphisms were observed.

**Conclusion:**

These findings imply that *ATBF1-A *mRNA levels are regulated at the transcriptional stage, but not by genetic mechanisms, deletions (LOH), or mutations.

## Background

Previous studies, including ours, have shown that loss of heterozygosity (LOH) on the long arm of chromosome 16 is one of the most frequent genetic events in breast, gastric and prostate cancers, implying the presence of one or more tumor suppressor genes (TSGs) at this location [1-7]. In breast cancer, the gene encoding *E-cadherin *at 16q22.1 was identified as a TSG, but only in the histological subgroup of lobular carcinoma [8]. Recently, the *AT-motif binding factor 1 *(*ATBF1*)-*A *gene (GenBank: L32832), which has been assigned to chromosome 16q22.3-23.1 [9], was identified as a reasonable candidate for tumor suppressor activity in solid tumors, based on its functional inhibition of cell proliferation and high rate of mutations in prostate cancer [10].

*ATBF1-A *was originally identified as a negative transcriptional factor for the *alpha-fetoprotein *(AFP) gene through binding with the AT-rich motif in the AFP enhancer element I [11,12]. In gastric cancer, absence of *ATBF1-A *is a distinct feature of AFP-producing cancer cells, which are characterized by a high malignant potential [7,13]. Moreover, *ATBF1-A *negatively regulates the c-Myb oncoprotein [14] and transactivates the cell cycle inhibitor *CDKN1A *[15]. Therefore, the *ATBF1-A *gene is considered to be a good TSG candidate in solid tumors.

Previously, we reported that reduced *ATBF1-A *mRNA levels in tumors correlated with axillary lymph node metastasis and estrogen receptor (ER)-α negative status in breast cancer, and with a worse prognosis [16]. Sun et al. confirmed the presence of reduced *ATBF1-A *mRNA levels in breast cancer cell lines [17]. However, the reduced *ATBF1-A *mRNA expression was attributed neither to promoter methylations nor to frequent somatic mutations [17]. Therefore, the authors concluded that *ATBF1-A *plays a role in breast cancer through transcriptional down-regulation rather than promoter methylation or mutations.

In addition to promoter methylations or mutations, LOH resulting from a deletion spanning one or more genes is one of the mechanisms by which the function of genes is lost. However, there are no papers in which has been reported the associations between LOH at the *ATBF1-A *locus [10] in the 16q22 minimal region and *AFBF1-A *mRNA levels, or between LOH at this locus and the clinicopathological factors in breast cancer. We performed LOH analysis at the 16q22 minimal region and mutational analysis focusing on specific loci in the *ATBF1-A *gene, which have been reported previously in prostate cancer[10]. Our analysis shows that *ATBF1-A *mRNA levels are not regulated by genetic machinery, LOH, or mutations. These findings could support the view that the *ATBF1-A *gene plays a role in breast cancer through transcriptional down-regulation rather than through LOH and mutations.

## Methods

### Patients and samples

Specimens of primary breast carcinomas were obtained by surgical excision from 127 female patients who received treatment at the Department of Breast and Endocrine Surgery, Nagoya City University Hospital, Nagoya, Japan, between 1993 and 2000. All the patients in the present study had *ATBF1-A *mRNA profiles, as reported previously [16]. Informed consent was obtained from all the patients before surgery. The ethics committee of Nagoya City University Graduate School of Medicine, Nagoya, approved the study protocol. Of the 127 tumors obtained from these patients, 109 were infiltrating ductal carcinomas and 14 were special type carcinomas, including 8 infiltrating lobular carcinomas. The remaining 4 patients had noninvasive carcinomas. The median age of the patients was 58 years (range, 34–88 years). The patients' tumors were classified according to the staging system of International Union Against Cancer as follows: 4 cases were classified as stage 0, 29 as stage I, 78 as stage II, 12 as stage III, and 4 as stage IV. Patients were graded histopathologically according to the modified Bloom and Richardson method proposed by Elston and Ellis [18]. Blood samples were also taken from each patient. Genomic DNA from the breast cancer specimens and blood samples were extracted by standard techniques. Breast cancer specimens were verified not to contaminate normal cells at 10% or more of each sample on the hematoxylin and eosin stained slides as previously described[19].

### Microsatellite markers and LOH analysis

**Figure 1:**
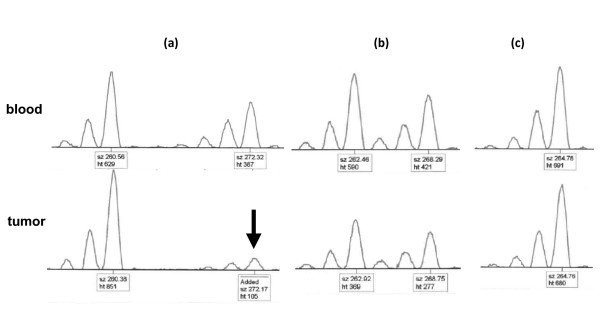
**Representative examples of microsatellite analysis for D16S3018 in breast cancer patients with paired tumor-blood samples**. **(a) **Loss of heterozygosity at D16S3018. The arrow indicates the allele loss. **(b) **Retention of heterozygosity. **(c) **Uninformative sample due to the homozygosity at D16S3018. Each peak has a box that provides the fragment size and peak height (upper and lower labels, respectively).

The breast cancer specimens as well as the autologous blood samples were screened using 6 polymorphic microsatellite markers spanning chromosome band 16q22 containing the *ATBF1-A *gene. The order of microsatellite loci was assessed as reported by Sun et al[10] and with reference to NCBI [20]. Details of the investigated markers and their chromosomal locations are provided in Table 1. Assays were performed by fluorescent-labeled PCR amplification using fluorescent dye-labeled forward primer and unlabeled reverse primer (Applied Biosystems). PCR was performed in 15-μL reaction volumes containing 120 ng of genomic DNA, 9 μL of ABI Prism True Allele PCR Premix (Applied Biosystems), 5 pmol of fluorescent dye-labeled forward primer, and 5 pmol of unlabeled reverse primer under the following cycling conditions recommended by manufacture's instructions: denaturation at 95°C for 12 min; 10 cycles at 94°C for 15 s, 55°C for 15 s, and 72°C for 30 s; 20 cycles at 89°C for 15 s, 55°C for 15 s, and 72°C for 30 s; and a final extension at 72°C for 10 min. Each 1-μL sample of the resulting PCR products was diluted with 20 μL of H_2_O, and a 1.0-μL aliquot of each diluted fluorescent-labeled PCR product was combined with 12 μL of formamide and 0.5 μL of GeneScan 500 LIZ size standard (Applied Biosystems). Capillary electrophoresis was then performed using an ABI 310 DNA Analyzer, and the results were analyzed using GeneMapper software (Applied Biosystems). Representative results are shown in Figure 1. Allelic loss at each microsatellite locus was considered to be present in tumor samples' DNA when there was at least a 65% peak reduction at one of a pair peak compared with the corresponding peak of normal DNA.

**Table 1:** Characteristics of polymorphic loci at the 16q22 minimal region

No.	Locus name	Product size (bp)	Genetic map (bp)	Genetic map (cM)	Microsatellite status
1	D16S3031	258–280	64338900–64339200	85.34	Heterozygous
2	D16S752	101–129	69892700–69892800	87.06	Heterozygous
3	D16S3106	166–206	70745300–70745500	88.18	Heterozygous
4	RH69880	149	71553100–71553200	ND	Homozygous
5	D16S2859	186	71675300–71675400	ND	Homozygous
6	D16S3018	244–270	72730200–72730500	90.65	Heterozygous
7	D16S3049	233–255	77478800–77479100	97.03	Heterozygous
8	D16S504	149	77723500–77723600	101.23	Heterozygous

The genetic maps were cited from NCBI Map Viewer . ND, not described.

### Gene alterationanalysis of *ATBF1-A *gene

With intent to analyze *ATBF1-A *mutations, we selected all the mutational spots in exon 9 and the most frequent deletion spot in exon 10 reported in prostate cancer because > 80% of the mutations reported in the paper were located in these spots (Table 2) [10]. Genomic DNA was amplified by PCR using the amplifying and sequencing primers for these spots on the *ATBF1-A *gene, as listed in Table 2. For mutation identification, the samples were subsequently analyzed using an ABI PRISM 310 Genetic Analyzer (Applied Biosystems). The sequencings from 50 to 250 bp, counting from the starting point, were regarded as the region of interest.

**Table 2:** Paired primers used to amplify the specific sequence of ATBF1-A, including the partial mutation loci previously reported in prostate cancer

				Primer sequence (5'→3')		
					
Exon	Mutation no.	Nucleotide*	Amino acid	Forward	Reverse	Product size (bp)
9	1	(5556–5559) del	Frameshift	AGCAACCGGTCAGCCAGAAC	GGTGGCATCCCTACACTCTCAG	336
	2	5602C→T	No change			
	3	(5891–5896) del		CTCCAAGCTCTAACTTACTAAGCCAA	AGTTGCAGCAGGGTCTCAGTT	349
	4	5897insCAA	Gln1741ins			
	5	6350delAG	Frameshift	AGCCAGCACCCCGAAAAGA	CTTGCCGCAGGAGTCACAC	360
	6	6447delG	Frameshift			
	7	7154C→T	R2160W	CACCGGCCCAGCCATCAGT	AACCCGGACTTGTCTGCCATCT	322
	8	8364A→G	Gln2564Arg	TCAACTCCTCAACAGCTCGCAA	GCTTGCACTGGCCTTTTCCTC	333
	9	8919C→T	A2749V	CGCAGGCCCACAGGAGA	CAAAGTCTTCAATCCCTTCCACC	321
	10	9380A→G	S2903G	AACTCTTCTAAGCCCTTCCTCCAT	CACTGTAGTCCACTGTACCTTCATTG	356
	11	9434G→A	A2921T	CCGAGCTTTTATAGCAAGGAATATG	ACTTGGACTTCTTTTCTTTTGCCC	322
10	12	(10814–10834)del	7 amino acid loss	TCCTTACTTTGTACCAGGCTTTTCTC	TCTTCTGGTTTGGGGGATTCTTTG	345
	13	(10814–10837)del	8 amino acid loss			
	14	(10826–10846)del	7 amino acid loss			

*ATBF1 mutations previously reported in prostate cancer by Sun et al. [10]

### Immunohistochemical staining of ER-α, progesterone receptor, and human epidermal growth factor receptor 2

Immunostaining of ER-α, progesterone receptor (PR), and human epidermal growth factor receptor 2 (HER2) was performed, as described previously [21]. Primary antibodies included monoclonal mouse antihuman estrogen receptor antibody (1D5; DAKO, Glostrup, Denmark) at 1:100 dilution for ER-α, antihuman PR antibody (PR636; DAKO, Kyoto, Japan) at 1:100 dilution for PR, and rabbit antihuman c-erbB2 oncoprotein antibody (DAKO) at 1:200 dilution for HER2. A Streptavidin-biotin system (SAB-PO kit; Nichirei, Tokyo, Japan) was used for the detection of ER-α, PR, and HER2 according to the manufacturer's instructions. The expression of ER-α and PR was scored by assigning a proportion score and an intensity score according to Allred's procedure [22]. Immunohistochemical scoring of HER2 was accomplished as described previously [21].

### Statistical analysis

The nonparametric Mann-Whitney *U*-test was used for statistical analysis of the association between LOH status at the *ATBF1-A *locus and *ATBF1-A *mRNA levels. The χ^2 ^test was used to compare LOH status at the *ATBF1-A *locus with the clinicopathological characteristics. All values with *P *< 0.05 were considered statistically significant.

## Results

### Loss of heterozygosity at chromosome band 16q22

**Figure 2:**
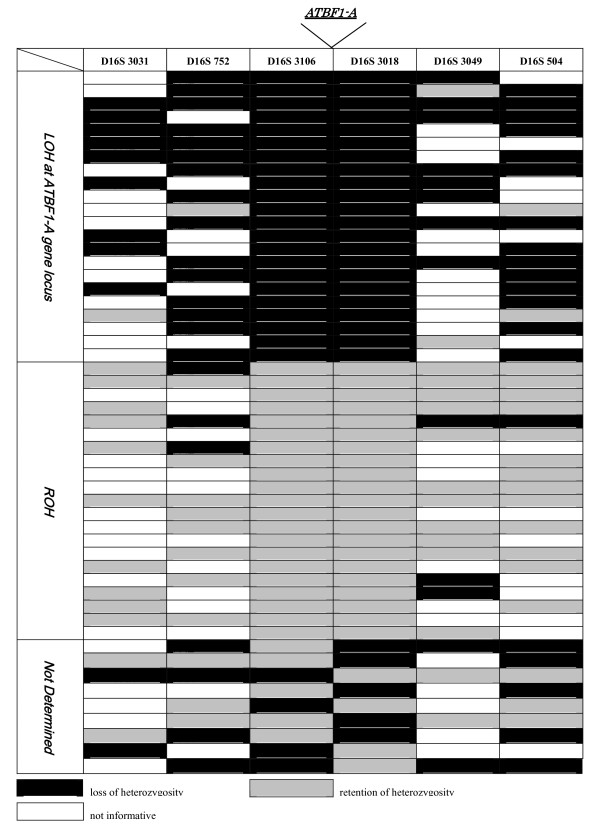
**Loss of heterozygosity (LOH) mapping of the 16q22 minimal region in breast cancer**. All cases except those that were uninformative are shown. The LOH status at the *ATBF1-A *gene locus was categorized as follows: LOH, indicating that LOH status at both D16S3106 and D16S3018 showed LOH; retention of heterozygosity (ROH), indicating that both D16S3106 and D16S3018 showed ROH; not determined, indicating that there was a discrepancy in LOH status between D16S3106 and D16S3018.

Initially, we performed LOH analysis using microsatellite markers spanning 16q22. Sun et al. narrowed the region of deletion at 16q22 in prostate cancer to 861 kb containing the *ATBF1-A *gene[10]. We used microsatellite markers either in the narrowed 861-kb region or close to the narrowed region spanning 16q22 (Table 1). However, both microsatellite markers in the narrowed region, RH69880 and D16S2859, were homozygous (uninformative). Thereafter, we performed LOH mapping using 6 other microsatellite markers. Next, to determine the most reasonable set of microsatellite markers reflecting LOH status at the *ATBF1-A *locus, we tested various kinds of microsatellite marker sets from among the 6 microsatellite markers (data not shown). Ultimately, we defined LOH status at the *ATBF1-A *locus as follows: LOH was identified when the microsatellite markers at both D16S3106 and D16S3018 showed LOH; retention of heterozygosity (ROH), when both D16S3106 and D16S3018 showed ROH; not determined (ND), when there was discrepancy in LOH status between D16S3106 and D16S3018, because *ATBF1-A *is located between these markers, LOH status at *ATBF1-A *is not rigorously determined in this case; and uninformative (UI), when there was constitutional homozygosity or an uninterpretable result for at least one of the two microsatellites. The partial results (all cases except UI) of LOH analysis at the 16q22 minimal region are shown in Figure 2. Constitutional heterozygous (informative) status in both D16S3106 and D16S3018 was observed in 52 cases (44.4%). Of the 52 cases, clear LOH was seen in 22 cases (42.3%), ROH in 21 (40.4%), and ND in 9 (17.3%). Subsequently, we analyzed the relationship between LOH status at the *ATBF1-A *locus and *ATBF1-A *mRNA expression levels as well as the clinicopathological factors in a study cohort consisting of these 43 informative cases, classifying them into LOH (n = 22) and ROH (n = 21) groups.

### Comparison of ATBF1 mRNA expression according to LOH status at the *ATBF1-A *locus

**Figure 3:**
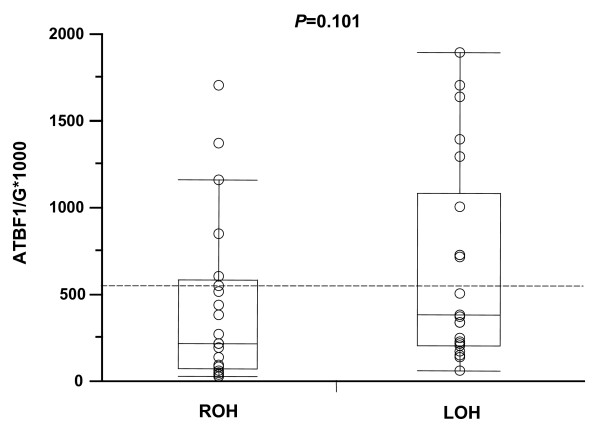
**Comparison of ATBF1-A mRNA levels according to loss of heterozygosity (LOH) status at the *ATBF1-A *locus**. There was no significant correlation between *ATBF1-A *mRNA levels and LOH status at the *ATBF1-A *locus (*P *= 0.101). ROH, retention of heterozygosity.

In a previous study, we demonstrated that the *ATBF-A *mRNA expression level was a significant prognostic marker in breast cancer [16]. However, the factors regulating the transcriptional level of the *ATBF1-A *gene are still not clear. Using *ATBF1-A *mRNA data, as reported previously [16], we investigated whether LOH status at the *ATBF1-A *locus regulates the transcriptional level of the *ATBF1-A *gene. Figure 3 shows a quantitative assessment of *ATBF1-A *mRNA expression according to LOH status at the *ATBF1-A *locus and demonstrates that there was no relationship between them. This finding indicates that another mechanism is involved in regulating the transcriptional level of the *ATBF1-A *gene.

### *ATBF1-A *nucleotide alterations in breast cancer

We next performed mutational analysis of the *ATBF1-A *gene to inspect whether *ATBF1-A *mRNA expression levels could be related with mutations succeeding to the instabilities of their transcripts. We selected all the mutational spots located in exon 9 and the most frequent deletion spot in exon 10 (Table 2), because > 80% of the mutations in the coding region of *ATBF1-A *gene reported in prostate cancer occurred in these locations. Twelve patients with tumors that did not demonstrate LOH at the *ATBF1-A *locus but with lower *ATBF1-A *mRNA expression were chosen as study subjects for mutational analysis. Overall, five sequence alterations, including both mutations and polymorphism, were found in the abovementioned spots in the 12 samples from these patients. Two patients had a somatic (blood sample not matched) 5602C > T mutation in one allele that did not involve amino acid substitution (i.e., synonym mutations). On the other hand, one patient had a 5602C > T blood-sample matched alteration in one allele that was reported on the dbSNP database [23]. We therefore regarded it as a single nucleotide polymorphism (SNP), as reported by Sun et al[17]. Both the remaining samples demonstrated a germ line alteration (blood-sample matched) in one allele. One of them, 8387A > G, caused a substitution of methionine for valine at codon 2572. The other alteration was a deletion (10799–10804) without frameshift. Although both gene alterations were not reported in the NCBI dbSNP and JSNP databases [24], we could not immediately rule out the possibility that these alterations were benign polymorphisms.

### Relationship between LOH status of the *ATBF1-A *locus and clinicopathological factors

As the reduced *ATBF1-A *mRNA expression levels correlated with unfavorable characteristics of tumors, ER-α- negative and lymph node metastasis, in our previous study[16], we wondered whether LOH status at *ATBF1-A *locus were related with clinicopathological factors as well. The results of this analysis are shown in Table 3. LOH at the *ATBF1-A *locus correlated with positivity of PR (*P *= 0.013) and negativity of HER2 (*P *= 0.024) status. Patients with ER-α-positive tumors were more likely to demonstrate LOH at the *ATBF1-A *locus than those with ER-α-negative tumors; however, the difference was not significant. Furthermore, the patients with LOH at the *ATBF1-A *locus more often had axillary lymph node involvement than those without LOH (*P *= 0.084). No relationship was observed between LOH at the *ATBF1-A *locus and other clinicopathological factors, such as age, tumor size, and histological grade.

**Table 3:** Correlation between clinicopathological factors and the LOH status at the 16q22 minimal locus

Factor	Loss of heterozygosity	Retention of heterozygosity	Total	*P*
Total	22 (51.2%)	21 (48.8%)	43 (100%)	
Age (year)				
≤ 50	7 (43.8%)	9 (56.2%)	16	NS
> 50	15 (55.6%)	12 (44.4%)	27	
Tumor size				
< 2.0	6 (46.2%)	7 (53.8%)	13	NS
≥ 2.0	16 (55.2%)	13 (44.8%)	29	
Not measurable	0	1	1	
Lymph node				
Negative	9 (37.5%)	15 (62.5%)	24	0.084
Positive	11 (64.7%)	6 (45.3%)	17	
Unknown	2	0	2	
Histological grade				
1/2	6 (60.0%)	4 (40.0%)	10	NS
3	14 (50.0%)	14 (50.0%)	28	
Unknown	2	3	5	
Estrogen receptor-α				
Negative	4 (40.0%)	6 (60.0%)	10	NS
Positive	17 (54.8%)	14 (45.2%)	31	
Unknown	1	1	2	
Progesterone receptor				
Negative	2 (18.2%)	9 (81.8%)	11	0.013*
Positive	16 (61.5%)	10 (38.5%)	26	
Unknown	4	2	6	
HER2				
Negative	18 (60.0%)	12 (40.0%)	30	
Positive	2 (20.0%)	8 (80.0%)	10	0.024*
Unknown	2	1	3	

Estrogen receptor-α, progesterone receptor, and human epidermal growth factor (HER) 2 were determined histochemically.

*P < 0.05.

## Discussion

Using polymorphic microsatellite markers spanning chromosome 16q22, we performed fluorescent-labeled PCR amplification and capillary electrophoresis to investigate LOH status at the 16q22 minimal region in paired specimens of blood and primary breast tumors from 127 patients. We assessed the relationship between LOH status at the *ATBF1-A *locus and mRNA levels of *ATBF1-A*, as well as the clinicopathological factors in breast cancer. Quantitative assessment of *ATBF1-A *mRNA expression according to LOH status at the *ATBF1-A *locus demonstrated no relationship between these factors. This finding completely consists with the result repoted by Kim et al., studied in hepatocellular carcinoma (HCC)[25]. We therefore concluded that a mechanism other than LOH was involved in regulating the transcriptional level of the *ATBF1-A *gene in breast cancer as like in HCC.

Accordingly, we screened mutations of the specific loci in the *ATBF1-A *gene that have previously been reported in prostate cancer [10]. The 12 tumors investigated for mutations had been predicted to have a higher frequency of gene alterations in the *ATBF1-A *gene, because they demonstrated lower *ATBF1-A *mRNA levels but showed ROH at the *ATBF1-A *locus (data not shown). In the mutational analyses, although none of the 12 samples studied showed somatic mutations with substitution of amino acids or frameshift, two germ line gene alterations were recorded. One of these alterations predicted a substitution of methionine for valine at codon 2572. This alteration also reported in gastric cancer as a somatic mutation at one allele, while the other allele had lost[7]. Although the gene alteration was not reported in the NCBI SNP and JSNP databases, pathogenic significance of the gene alteration was also not verified by functional assay. The remaining gene alteration produced a 6-nucleotide deletion without frameshift in glutamic acid rich domain in exon10. Although the 3 – 24 nucleotides deletions are prevalent in various kind of tumors[6,7,10,26], its pathogeny is controversial among those tumors. In prostate cancer, Xu et. reported the deletions in germline and its relevance with the susceptibility to prostate cancer[26]. Contrast to that, in breast cancer, Cleton-Jansen et al. reported no association between these germline deletions and suscetability to breast cancer[6]. This indicate that these germline deletions has different impacts in the suscetabilities to these tumors. As in the present study, the deletion was also germline, they might be benign polymorphisms regarding with the susceptibility to breast cancer.

A larger case-control study compared between cancer patients and healthy individuals and functional analysis in each gene alteration should be performed to reveal their pathogenic significances in tumorigenesis. As, consequently, somatic mutations with substitution of amino acids or frameshift were not seen in these samples, we concluded that *ATBF1-A *mRNA levels may be regulated at the transcriptional stage, but are not regulated by genetic mechanisms, deletions (LOH), or mutations in breast cancer. Infrequent somatic mutations in *ATBF1-A *gene have previously been reported, except for prostate cancer. The frequencies were 8.6% in gastric cancer[7], 0% in HCC[25], 0% in breast cancer[6]. In addition, Sun et al. and Kim et al. reported infrequent methylation at *ATBF1 *gene promoter in breast cancer and HCC, respectively[17,25]. Based on these reports, we speculate that the methylations at ATBF1-A gene are also not attributed to the downregulation of ATBF1-A transcripts, though we did not performed the methylation analysis.

Recently, posttranscriptional mRNA repression associated with microRNA (miRNA) have been discussed as an alternative mechanism of mRNA modulation at the posttranscriptional level in mammals as well as metazoa [27]. According to the prevailing model, posttranscriptional repression by miRNA is determined by the complementarity to the miRNA of the target mRNA[27]. Therefore, we may speculate that posttranscriptional cleavage of mRNA by miRNA-associated machinery is the molecular mechanism of *ATBF1-A *mRNA regulation. This may explain why there is a discrepancy between LOH status at *ATBF1-A *locus and *ATBF1-A *mRNA levels.

LOH status at the *ATBF1-A *locus significantly correlated with positivity of PR (*P *= 0.013) and negativity of HER2 (*P *= 0.024) status. Similar results were previously reported by Wang [28], who found that 16q23-24 genetic loss significantly correlated with ER-α positivity and HER2 negativity. These biological features, hormonal receptor positivity, and HER2 negativity, are reminiscent of the "luminal-type" tumors described by Perou et al. [29]. Based on the study by Wang [28] and the present study, a target gene at the narrowed locus spanning from 16q22 to 16q24 may determine the biological features of the luminal-type. Recent cytogenetic approaches, such as comparative genomic hybridization[30], may help reveal the new target gene at this locus in such cohorts.

## Conclusion

Using polymorphic microsatellite markers spanning chromosome band 16q22, we defined LOH status at the *ATBF1-A *locus and performed comparative analysis between LOH and ROH groups. However, we did not find a significant correlation between LOH status at the *ATBF1-A *locus and *ATBF1-A *mRNA levels. Furthermore, we found no somatic mutations with amino acid substitution in 12 tumor samples from selected patients who were predicted to have a higher frequency of gene alterations, although two germ line alterations with the possibility of polymorphism were noted. We therefore conclude that *ATBF1-A *mRNA levels may be regulated at the transcriptional stage.

## Abbreviations

LOH: loss of heterozygosity; *ATBF1-A*: AT-motif binding factor 1-A; TSG: tumor suppressor gene; AFP: alpha-fetoprotein; ROH: retention of heterozygosity; ND: not determined; UI: uninformative; SNP: single nucleotide polymorphism; miRNA: microRNA

## Competing interests

The authors declare that they have no competing interests.

## Authors' contributions

K.K. performed the molecular genetic studies, participated in the LOH analysis and sequence alignment, and drafted the manuscript. Z.Z. performed the sequence alignment and scored the status of immunohistochemical staining. H.Y. was involved in the study design and scored the status of immunohistochemical staining. Y.M. was involved in the study design and assisted in drafting of the manuscript. Y.Y. and H.I. conceived the study, participated in its design and coordination, and assisted in drafting of the manuscript. All authors have read and approved the final manuscript.

## Pre-publication history

The pre-publication history for this paper can be accessed here:


